# Validation of a parent proxy-reported beverage screener compared to a 24-hour dietary recall for the measurement of sugar-containing beverage intake among young children

**DOI:** 10.1371/journal.pone.0288768

**Published:** 2023-07-20

**Authors:** Isobel Sharpe, Sharon I. Kirkpatrick, Brendan T. Smith, Charles D. G. Keown-Stoneman, Jessica A. Omand, Shelley Vanderhout, Christine Warren, Jonathon L. Maguire, Catherine S. Birken, Laura N. Anderson, on behalf of the TARGet Kids! collaboration

**Affiliations:** 1 Department of Health Research Methods, Evidence and Impact, McMaster University, Hamilton, Ontario, Canada; 2 School of Public Health Sciences, University of Waterloo, Waterloo, Ontario, Canada; 3 Department of Health Promotion, Chronic Disease and Injury Prevention, Public Health Ontario, Toronto, Ontario, Canada; 4 Division of Epidemiology, Dalla Lana School of Public Health, University of Toronto, Toronto, Ontario, Canada; 5 Applied Health Research Centre of the Li Ka Shing Knowledge Institute of St. Michael’s Hospital, Unity Health, Toronto, Ontario, Canada; 6 Division of Biostatistics, Dalla Lana School of Public Health, University of Toronto, Toronto, Ontario, Canada; 7 Division of Child Health Evaluative Sciences (CHES), Sick Kids Research Institute, Toronto, Ontario, Canada; 8 Faculty of Medicine, Department of Nutritional Sciences, University of Toronto, Toronto, Ontario, Canada; 9 Department of Pediatrics, St. Michael’s Hospital, Unity Health, Toronto, Ontario, Canada; MRC Unit The Gambia at LSHTM, GAMBIA

## Abstract

Measures that can provide reasonably accurate estimates of sugar-containing beverage (SCB) intake among children are needed. The primary objective of this study was to evaluate the relative validity of a short beverage screener (Nutrition and Health Questionnaire, NHQ) compared to a 24-hour recall (Automated Self-Administered 24-h (ASA24) Dietary Assessment Tool-Canada) for assessing parent proxy-reported daily SCB intake among children aged 4–14 years from the TARGet Kids! research network in Toronto, Canada. Children for whom a NHQ completed between March 2018 and June 2019 and an ASA24 completed within one year were included. A total of 471 parents who completed the NHQ beverage screener were also asked to complete the ASA24. One-hundred sixty-three completed the ASA24 and of this group, 109 were analyzed. Estimates of daily intake of 100% juices, sweetened drinks and soda, and total SCBs from the two measures were compared. The mean difference in beverage intake, Spearman correlations, and Bland-Altman plots were estimated for continuous measures. The kappa coefficient, sensitivity, and specificity were calculated for dichotomous measures of any daily intake versus none. The mean difference in total SCB intake between the NHQ and ASA24 was 0.14 cups/day (95% CI 0.01, 0.29) and the correlation was 0.43 (95% CI 0.26, 0.57). Sensitivity and specificity for any daily SCB intake were 0.63 and 0.76, respectively. Overall, parent proxy-reporting of children’s total SCB intake from a beverage screener can provide reasonable estimates of SCB intake when detailed dietary assessment is not feasible.

## Introduction

Although childhood sugar-containing beverage (SCB) consumption has stabilized over recent decades, intake remains high [1–3]. SCB consumption has been associated with weight gain and obesity [4–6], cardiovascular risk factors [7–9], and type 2 diabetes [10]. Consequently, assessment of SCB intake among children is important for informing chronic disease prevention. However, in many contexts it is not feasible to conduct detailed dietary assessment and therefore rapid measures providing reasonable estimates of SCB intake are important for improving the validity of SCB studies. Due to their ease of administration and low respondent burden [11], parent proxy-reported short dietary screeners may be useful for assessing SCB intake in children. Previous studies evaluating the validity of SCB screeners compared to comprehensive measures of dietary intake, including 24-hour recalls and food records, have focused on child and adolescent self-reporting populations, where findings have been mixed [12–19]. Few studies have evaluated parent proxy-reporting to assess the validity of screeners measuring SCB intake among young children [20,21]. Further, the screeners used in these studies focused on obesity-related foods and only contained one to two SCBs [20,21]. Previous studies were also limited in their methods of assessing validity based on best practice guidelines [22].

The primary objective of this study was to evaluate the relative validity of a brief dietary screener completed by a parent proxy for estimating daily SCB intake among children aged four years and older compared to a 24-hour recall. The secondary objective was to evaluate the agreement between dichotomous measures of any daily intake of SCBs versus none.

## Materials and methods

### Study design and participants

A validation study was conducted using existing data from The Applied Research Group for Kids (TARGet Kids!) cohort study following best practice guidelines for validating dietary assessment methods [22]. TARGet Kids! is a primary care pediatric network established in 2008 [23]. Children <6 years of age are recruited from primary care pediatric and family medicine practices in the Greater Toronto Area, Ontario, Canada and followed prospectively into adolescence. Children are excluded at recruitment if they had health conditions affecting growth (e.g. failure to thrive, cystic fibrosis), severe acute or chronic conditions or developmental delay (other than asthma and high functioning autism) and if families are unable to complete English questionnaires [23].

Children were included in the present validation study, which was nested within TARGet Kids!, if they had a TARGet Kids! visit (either baseline or follow-up) between March 2018-June 2019 and were ≥4 years of age. Additionally, parents must have completed the TARGet Kids! Nutrition and Health Questionnaire (NHQ), which included a beverage screener, and a 24-hour recall for their child within one year of the NHQ to facilitate comparison between the two measures. The age cut-off for our sample was selected in order to avoid overburdening the parents of the younger children in the TARGet Kids! program, who regularly complete a number of other age-specific questionnaires. No upper age limit was implemented.

To capture child demographic characteristics, we used parent-completed survey data from TARGet Kids! that included child age and sex, maternal ethnicity, and family income. Child height and weight were measured by trained research assistants and body mass index z-scores were calculated based on the recommended World Health Organization Standards and Reference [24].

Parents provided written consent for participation and research ethics board approval was obtained from the Hospital for Sick Children, Unity Health, Toronto Ontario and the Hamilton Integrated Research Ethics Board.

### Short beverage screener

The NHQ short 12-item beverage screener asked parents to “circle how many cups of each drink your child has currently in a typical day, (1 cup = 8 ounces = 250 ml)” with response options ranging from 0 to 5+ cups per day (S1 Fig). Milk or milk substitutes (e.g., cow’s milk, infant formula, soy milk) and tea were excluded from our SCB definition because of insufficient detail on whether they included added sugars. Therefore, we included the three remaining beverage categories as SCBs: 100% juice (e.g., apple, orange), sweetened drinks (e.g., Kool aid, Sunny D), and soda or pop. The NHQ beverage screener is intended to estimate typical absolute beverage intake.

The NHQ beverage screener has been used since 2008 in the TARGet Kids! cohort and face validity has been informally evaluated by several registered dietitians and pediatricians as well as through feedback from parents. Further, responses from the beverage screener have been associated with outcomes in a manner consistent with *a priori* hypotheses [25,26], suggesting the measure may have construct validity. Relative validity (i.e., comparison to a reference method) has not been previously established.

### 24-hour recall (reference measure)

Measurement of children’s dietary intake using 24-hour dietary recalls was introduced to the TARGet Kids! protocol in March 2018. Recalls were administered to parents using the 2016 Automated Self-Administered 24-h (ASA24) Dietary Assessment Tool-Canada (https://epi.grants.cancer.gov/asa24/resources/), developed by the US National Cancer Institute [27] and adapted for use in Canada, including through a linkage to the Canadian Nutrient File [28]. Parents of eligible children were sent a standardized email requesting online completion of the ASA24-Canada-2016 from midnight-midnight on the previous day. If the recall was not completed, a reminder email was sent after one week and a second reminder two weeks later.

ASA24 uses a modified version of the United States Department of Agriculture’s Automated Multiple-Pass Method through a self-administered online interface [27]. Used in national surveillance, the Automated Multiple-Pass Method promotes reporting of all foods and beverages consumed the previous day through successive passes and prompts and has been successfully validated against objective measures of energy intake [29,30]. The ASA24 itself has been validated for use with adults in several studies using unbiased measures such as biomarkers and feeding studies [31–34], and using other measures such as interviewer-administered 24-hour recalls [35–37]. For this study, ASA24 data were linked by study identification number to the NHQ from the most recent TARGet Kids! visit. The data cleaning procedure has been described previously [38]. Briefly, data cleaning guidelines from the US National Cancer Institute were consulted [39]. Entries that were implausible (i.e., accidental parent self-report) or partially completed were removed.

### Quantifying beverage intake based on ASA24

Foods and beverages reported using ASA24 are autocoded using food codes from the Canadian Nutrient File. All SCB-related food codes were classified into one of three categories corresponding with those used by the NHQ beverage screener: 100% juice, sweetened drinks, and soda or pop. Beverage names were used to classify the SCB-related food codes, where beverages such as “fruit drink” and “apple juice with added sugar” were considered sweetened drinks, while items such as “apple juice” and “raw grapefruit juice” were considered 100% juice. The classification was also informed by an approach used in earlier work [40]. The final classifications were reviewed and consensus reached by two registered dietitians (JAO, SV). All beverages in the ASA24 were reported in grams, while the NHQ was reported in cups. To convert the ASA24 measures into their liquid equivalents, density was assumed at 1g/mL.

### Statistical analysis

Estimates of mean beverage volume (cups/day) based on the NHQ and ASA24 were compared for the following SCB categories: 100% juice, sweetened drinks, soda, and total SCBs (where total SCBs were the sum of the former). Soda and sweetened drinks were subsequently combined into one category due to small cell sizes when evaluated separately. Children with missing data for any of the SCB categories were removed from this analysis.

Descriptive statistics were used to characterize the study population, calculated as mean and standard deviation (SD) for continuous measures and as frequencies (%) for categorical measures. Mean volumes (cups/day) were calculated for both measures and group-level agreement was tested using the Wilcoxon signed-rank test. In conjunction, we used a Bland-Altman plot to examine the mean difference between intake estimates from the two measures. Spearman correlation coefficients were also calculated to determine the association between the two measures. Individuals were further classified using 2x2 tables for any daily SCB intake (>0 cups/day) compared to none (0 cups/day). The results were evaluated for agreement using the kappa coefficient, interpreted using guidelines from Landis and Koch [41]. Sensitivity and specificity were also calculated. As the data were not normally distributed, non-parametric tests were used. Statistical analysis was performed using SAS Studio 3.71 (SAS Institute Inc., Cary, NC, USA).

### Sensitivity analysis

A limitation of this study was that the NHQ and ASA24 captured different time periods; the NHQ captured “typical daily” beverage intake while the ASA24 captured intake from the previous day. To address this issue, we performed a sensitivity analysis of the children whose parents indicated that their ASA24 report was representative of their child’s usual intake, suggesting that the ASA24 may represent typical intake for this sub-sample.

## Results

**Fig 1:**
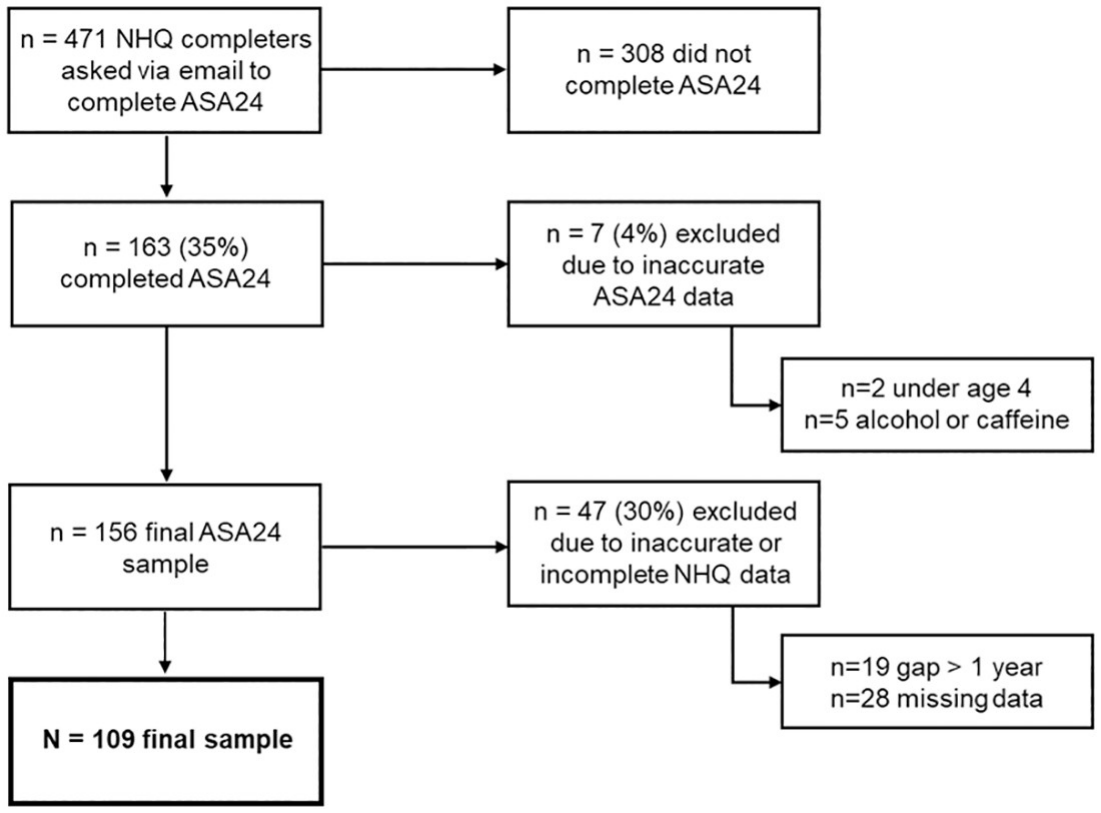
Study flow chart. Flow chart of children participating in the validation of a parent proxy-reported short beverage screener (Nutrition and Health Questionnaire; NHQ) against a 24-hour recall (Automated Self-Administered 24-h Dietary Assessment Tool-Canada; ASA24). Shows reasons for exclusion and final sample size.

During the study period, 471 parents who had completed the NHQ beverage screener were also asked to complete the ASA24 (Fig 1). A total of 163 (35%) completed the ASA24, of which seven were excluded for problems related to its completion (n = 2 under age four, n = 5 parents were suspected to have reported their own rather than the children’s intake), leaving 156 valid responses. Forty-seven (30%) additional responses were excluded for having a gap between completing the NHQ and ASA24 of greater than one year (n = 19) or for having missing NHQ data (n = 28). One hundred nine children made up the final sample. Table 1 describes the study population.

**Table 1:** Descriptive characteristics of study sample of N = 109 children participating in the validation of a parent proxy-reported short beverage screener against a 24-hour recall.

Characteristic	
	**Mean (SD)**
Child’s Age (years)	8.1 (SD 3.0)
Time between NHQ and ASA24 (months)	3.4 (SD 2.7)
	**n (%)**
Child’s Age (years)	
4–6 (n, %)	36 (33)
7–10 (n, %)	48 (44)
11–14 (n, %)	25 (23)
Child’s Sex	
Female	49 (45)
Male	60 (55)
Child’s Number of Siblings	
0	17 (21)
1	47 (57)
2 or more	19 (23)
Missing	26
Family Income	
Less than $30,000	1 (1.0)
$30,000 to $79,999	7 (6.7)
$80,000 to $149,999	35 (34)
$150,000 or more	61 (59)
Missing	5
Child’s Body Mass Index Z-Score	
≤1 (underweight or normal)	91 (84)
>1–2 (overweight)	13 (12)
>3 (obesity)	4 (3.7)
Missing	1
Maternal Ethnicity	
European	69 (69)
Asian	20 (20)
Other^a^	11 (11)
Missing	9

Values are reported as Mean (SD) or n (%) where appropriate.

^a^Includes Arab, African, Latin American, mixed ethnicity, and other.

**Fig 2:**
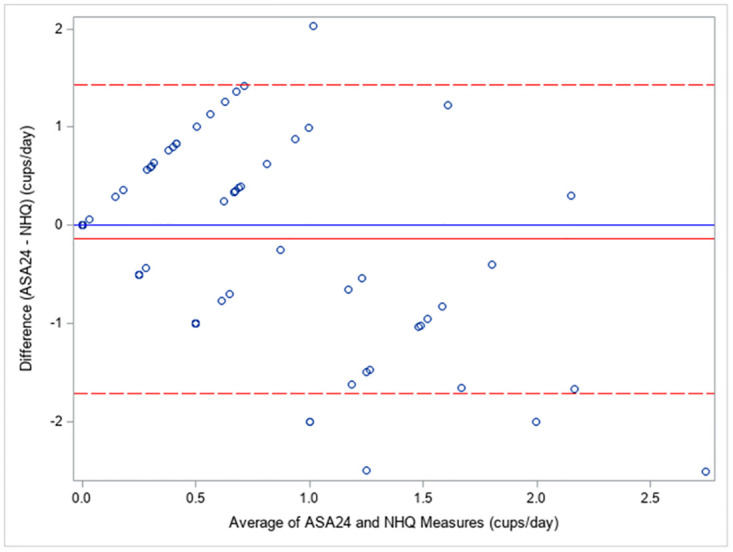
Bland-Altman plot. Describes the agreement between a parent proxy-reported short beverage screener (Nutrition and Health Questionnaire; NHQ) and a 24-hour recall (Automated Self-Administered 24-h Dietary Assessment Tool-Canada; ASA24) for total sugar-containing beverages (SCBs) among N = 109 children. The solid red line represents the mean of the difference between the two measures (-0.14 cups/day). The dashed red lines represent +/- 2 standard deviations of this difference (1.43 cups/day, -1.72 cups/day).

Table 2 shows the difference and correlation between the NHQ and ASA24 for mean beverage volume. The mean difference (NHQ–ASA24) for total SCBs was 0.14 (95% CI -0.01, 0.29) cups/day (Fig 2), 100% juice was 0.28 (95% CI 0.16, 0.40) cups/day, and sweetened drinks and soda was -0.14 (95% CI -0.23, -0.04) cups/day. Spearman correlations were 0.43 (95% CI 0.26, 0.57) for total SCBs, 0.51 (95% CI 0.36, 0.64) for 100% juice, and 0.26 (95% CI 0.07, 0.42) for sweetened drinks and soda.

**Table 2:** Mean difference and correlation between beverage volumes for the N = 109 children participating in the validation of a parent proxy-reported short beverage screener (Nutrition and Health Questionnaire; NHQ) against a 24-hour recall (Automated Self-Administered 24-h Dietary Assessment Tool-Canada; ASA24).

Beverage Group	Mean Beverage Volume NHQ in cups/day (SD)	Mean Beverage Volume ASA24 in cups/day (SD)	Mean Difference (NHQ–ASA24) cups/day (95% CI)	Wilcoxon Signed Rank Test of Difference	Spearman Correlation (95% CI)
**Total SCBs**	0.54 (0.85)	0.39 (0.57)	0.14 (-0.01, 0.29)	p = 0.078	0.43 (0.26, 0.57)
**100% Juice**	0.43 (0.70)	0.15 (0.32)	0.28 (0.16, 0.40)	p<0.0001	0.51 (0.36, 0.64)
**Sweetened Drinks + Soda or Pop**	0.11 (0.41)	0.25 (0.45)	-0.14 (-0.23, -0.04)	p = 0.0018	0.26 (0.07, 0.42)

Beverage volumes are reported as mean (SD). Mean difference and Spearman correlation are reported with the 95% confidence interval (CI).

Table 3 shows the 2x2 tables for the NHQ and ASA24 for the comparison of any daily intake (>0 cups/day) to none (0 cups/day). The kappa values were 0.39 (95% CI 0.22, 0.57) for total SCBs, 0.44 (95% CI 0.27, 0.61) for 100% juice, and 0.18 (95% CI -0.01, 0.37) for sweetened drinks and soda. Sensitivity ranged from 0.21 (for sweetened drinks and soda) to 0.83 (for 100% juice). Specificity was high for all SCB categories (≥ 0.74).

**Table 3:** Kappa coefficient, sensitivity, and specificity for any daily SCB intake (>0 cups/day) compared to none (0 cups/day) for the N = 109 children participating in the validation of a parent proxy-reported short beverage screener (Nutrition and Health Questionnaire; NHQ) against a 24-hour recall (Automated Self-Administered 24-h Dietary Assessment Tool-Canada; ASA24).

NHQ		ASA24		k (95% CI)	Sensitivity	Specificity
		Any	None			
**Total SCBs**	**Any**	29	15	0.39 (0.22, 0.57)	0.63	0.76
	**None**	17	48			
**100% Juice**	**Any**	19	22	0.44 (0.27, 0.61)	0.83	0.74
	**None**	4	64			
**Sweetened Drinks + Soda or Pop**	**Any**	6	5	0.18 (-0.01, 0.37)	0.21	0.94
	**None**	23	75			

Kappa statistic is reported with the 95% confidence interval (CI).

Ninety-three percent of parents (n = 101) indicated that their ASA24 report was representative of their child’s usual intake (S1 Table). Within this usual intake sub-sample, we observed findings similar to the main analysis (S2 and S3 Tables).

## Discussion

The aim of this study was to evaluate the relative validity of a short beverage screener compared to a 24-hour recall for assessing parent proxy-reported daily SCB intake among children aged 4–14 years (n = 109). Overall, we found moderate agreement between the two measures, supporting the potential utility of a short beverage screener for estimating SCB intake when detailed assessments are not available. This is particularly relevant to the context of TARGet Kids!, where a short beverage screener may be useful in reducing respondent burden in when parents are asked to complete a number of other measures.

In our study, the measurement of parent proxy-reported daily SCB intake in children aged 4–14 years from a beverage screener was moderately correlated with intake estimated using the ASA24 dietary recall. These findings are comparable to validation studies using similar parent proxy-reported measures, which reported Spearman correlations of 0.41 for 100% juice and sugar-sweetened beverages (single-item SCB screening question vs. NHANES questionnaire) [12] and 0.55 for sweetened beverages (Children’s Dietary Questionnaire, a short food frequency questionnaire, vs. 7-day food checklist) [21]. In contrast, a study of preschool-age children comparing the Eating and Physical Activity Questionnaire, a short questionnaire on physical activity and diet, with a 24-hour recall reported correlations of 0.88 for fruit juice and 0.82 for cordial/soft drinks [20]. Our study may have resulted in lower correlations than Bennett et al. [20] because they administered the two dietary measures consecutively, in contrast to our study where the time between the two measures was longer (mean time 3.4 SD 2.7 months). Our observed kappa coefficients ranged from 0.18–0.44, indicating slight-to-fair agreement between the NHQ and ASA24. This was comparable to a study of children 0–17 years of age that reported a kappa coefficient of 0.42 (95% CI 0.24, 0.60) for daily intake of sugar-sweetened beverages and 100% fruit juice [12].

Taken together, the results indicate that our short beverage screener produced reasonable agreement when compared to the ASA24 for total SCB intake. This measure may be feasible for rapidly assessing SCB intake among young children in situations where detailed dietary assessment is not realistic in terms of participant burden. However, it is important to recognize that all self-reported dietary intake measures are affected by measurement error, both random (e.g., estimates of usual intake being influenced by day-to-day variation) and systematic (e.g., biases related to recall, social desirability) [22]. This may be especially true for short screeners that do not capture total intake. Regarding the variation among categories, the 100% juice category showed strong agreement between the NHQ and ASA24. On the other hand, the agreement for sweetened drinks and soda or pop was poor. One contributing factor to this poor agreement may have been that our beverage screener did not differentiate between regular versus diet soda and parents may have reported these differently. Another factor may have been confusion surrounding the definition of a juice, which was considered to be a 100% fruit juice rather than a fruit-flavoured sweetened drink. Reporting may also be affected by social desirability bias if parents are more likely to report 100% juice compared to sweetened drinks. In future, qualitative methods such as focus groups may be useful for enhancing the clarity of the NHQ and improving its ability to effectively capture all SCB types.

Potential limitations of this study include the different time periods for each measure. While the NHQ captured “typical daily” beverage intake, the ASA24 captured intake from the previous day. Our sensitivity analysis of the 101 children reporting typical intake on the ASA24 showed largely similar results to our main analysis, suggesting that this may not have been an issue for our sample. However, our results may not be applicable to other populations such as those where parents are less aware of what their children consume throughout the day. For example, older children may be more likely to consume SCBs outside of the home environment, potentially resulting in a degree of misclassification within our sample.

Another limitation was that the NHQ and ASA24 were completed up to one year apart. Therefore, factors such as seasonality and child development may have contributed to differences between the measures. While it is a strength of this study that data from a 24-hour recall was available from ASA24, we were ultimately comparing two error-prone measures of SCB intake. Thus, our findings may have been artificially inflated by correlated errors [22] and not solely a result of concordant intake patterns. Lastly, the response rate for the ASA24 was low. This resulted in a small and somewhat homogeneous study sample, which was not reflective of the overall TARGet Kids! cohort [23]. SCB intake is often higher among children from non-white, low-income families [42], therefore the results of this study may not be generalizable. As discussed in our previous work [38], efforts are needed to increase response rates such as communication using multiple methods or the use of cash-based incentives.

## Conclusions

Overall, the comparison between the NHQ and ASA24 showed that parent proxy-reporting of children’s SCB intake as measured in a beverage screener was moderately agreeable when compared to 24-hour recall. However, agreement varied by beverage category. These findings support the potential utility of a brief screener but emphasize the need to recognize measurement error. Adjustment for measurement error or calibration sub-studies should be considered in studies evaluating the impact of SCBs on health outcomes when comprehensive dietary assessment is not feasible.

## Supporting information

S1 Fig Question number 28 on the *TARGet Kids*! Nutrition and Health Questionnaire (NHQ). This was the short beverage screener used in this study for comparison with the 24-hour dietary recall.
(TIF)

S1 Table Descriptive characteristics of a sub-sample of N = 101 children participating in the validation of a parent proxy-reported short beverage screener against a 24-hour recall. Sub-sample contained those children whose parents indicated that their 24-hour recall represented typical intake. Values are reported as Mean (SD) or n (%) where appropriate.
(DOCX)

S2 Table Mean difference and correlation between beverage volumes for a sub-sample of N = 101 children participating in the validation of a parent proxy-reported short beverage screener (Nutrition and Health Questionnaire; NHQ) against a 24-hour recall (Automated Self-Administered 24-h Dietary Assessment Tool-Canada; ASA24). Sub-sample contained those children whose parents indicated that their 24-hour recall represented typical intake. Beverage volumes are reported as mean (SD). Mean difference and Spearman correlation are reported with the 95% confidence interval (CI).
(DOCX)

S3 Table Kappa coefficient, sensitivity, and specificity for any daily SCB intake (>0 cups/day) compared to none (0 cups/day) for a sub-sample of N = 101 children participating in the validation of a parent proxy-reported short beverage screener (Nutrition and Health Questionnaire; NHQ) against a 24-hour recall (Automated Self-Administered 24-h Dietary Assessment Tool-Canada; ASA24). Sub-sample contained those children whose parents indicated that their 24-hour recall represented typical intake. Kappa statistic is reported with the 95% confidence interval (CI).
(DOCX)
