# Supplementary figures and images for: Validation of a parent proxy-reported beverage screener compared to a 24-hour dietary recall for the measurement of sugar-containing beverage intake among young children
Source: PLoS One. 2023 Jul 20;18(7):e0288768. doi: 10.1371/journal.pone.0288768 (PMC10358879; doi:10.1371/journal.pone.0288768)

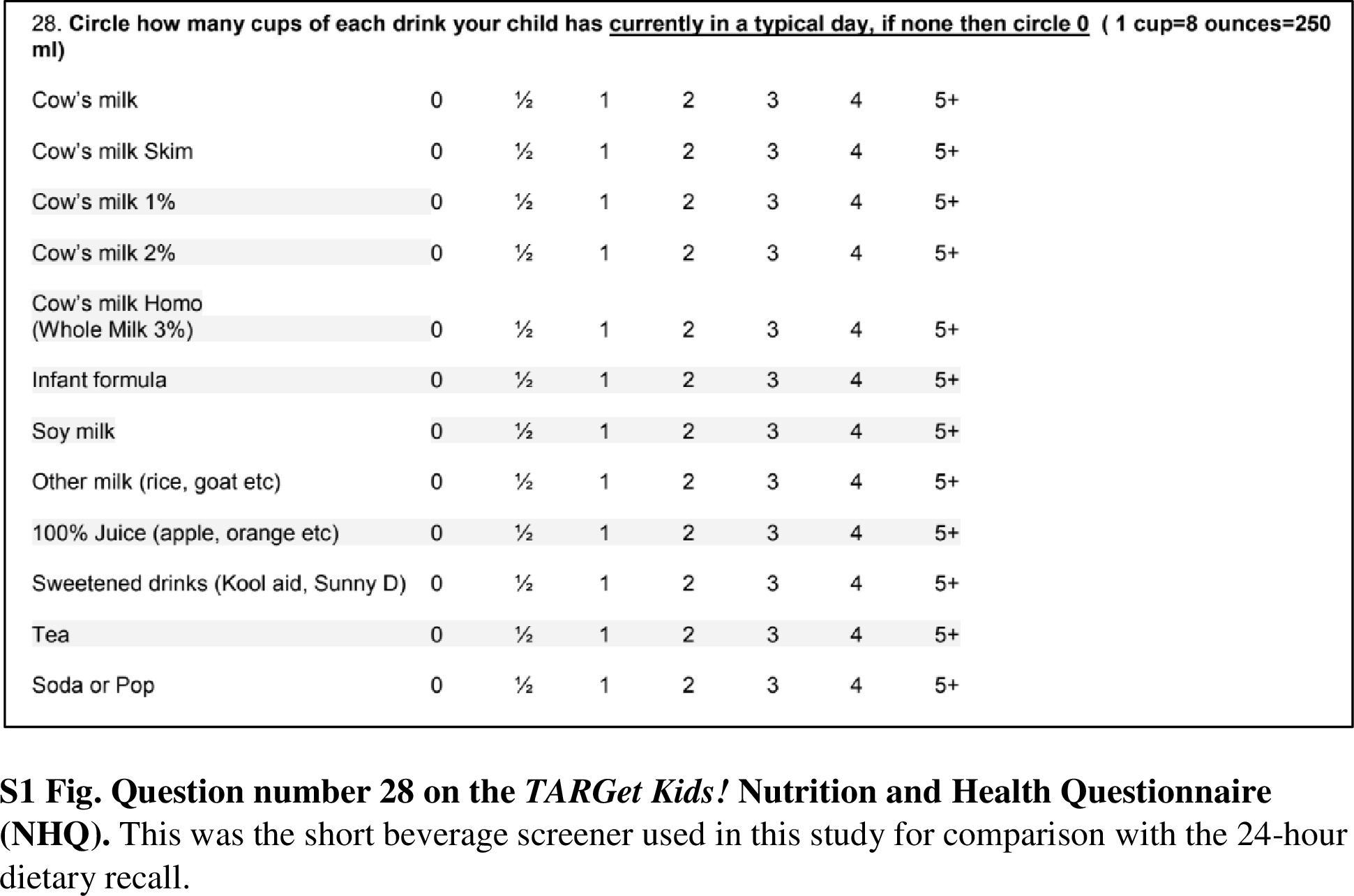

Supplement: S1 Fig — This was the short beverage screener used in this study for comparison with the 24-hour dietary recall. (TIF) [file pone.0288768.s001.tif]
