# Supplementary material for: Validation of a parent proxy-reported beverage screener compared to a 24-hour dietary recall for the measurement of sugar-containing beverage intake among young children
Source: PLoS One. 2023 Jul 20;18(7):e0288768. doi: 10.1371/journal.pone.0288768 (PMC10358879; doi:10.1371/journal.pone.0288768)
Supplement: S1 Table — Sub-sample contained those children whose parents indicated that their 24-hour recall represented typical intake. Values are reported as Mean (SD) or n (%) where appropriate. (DOCX) [file pone.0288768.s002.docx]

**S1 Table.** **Descriptive characteristics of a sub-sample of N=101 children participating in the validation of a parent proxy-reported short beverage screener against a 24-hour recall. Sub-sample contained those children whose parents indicated that their 24-hour recall represented typical intake.**

| **Characteristic** |  |
| --- | --- |
|  | **Mean (SD)** |
| Child Age (years) | 7.9 (SD 3.0) |
| Time between NHQ and ASA24 (months) | 3.2 (SD 2.7) |
|  | **n (%)** |
| Child Age (years)  4-6 (n, %)  7-10 (n, %)  11-14 (n, %) | 35 (35)  46 (46)  20 (20) |
| Sex  Female  Male | 46 (46)  55 (54) |
| Siblings  0  1  2 or more  Missing | 17 (22)  44 (58)  15 (20)  25 |
| Family Income  Less than $30,000  $30,000 to $79,999  $80,000 to $149,999  $150,000 or more  Missing | 1 (1.0)  7 (7.2)  30 (31)  59 (61)  4 |
| Body mass index z-score  ≤1 (underweight or normal)  >1-2 (overweight)  >3 (obesity)  Missing | 84 (84)  12 (12)  4 (4.0)  1 |
| Maternal Ethnicity  European  Asian  Other^a^  Missing | 65 (71)  20 (22)  7 (7.6)  9 |

Values are reported as Mean (SD) or n (%) where appropriate.

^a^Includes Arab, African, Latin American, mixed ethnicity, and other.
