# Supplementary material for: Validation of a parent proxy-reported beverage screener compared to a 24-hour dietary recall for the measurement of sugar-containing beverage intake among young children
Source: PLoS One. 2023 Jul 20;18(7):e0288768. doi: 10.1371/journal.pone.0288768 (PMC10358879; doi:10.1371/journal.pone.0288768)
Supplement: S2 Table — Sub-sample contained those children whose parents indicated that their 24-hour recall represented typical intake. Beverage volumes are reported as mean (SD). Mean difference and Spearman correlation are reported with the 95% confidence interval (CI). (DOCX) [file pone.0288768.s003.docx]

**S2 Table. Mean difference and correlation between beverage volumes for a sub-sample of N=101 children participating in the validation of a parent proxy-reported short beverage screener (Nutrition and Health Questionnaire; NHQ) against a 24-hour recall (Automated Self-Administered 24-h Dietary Assessment Tool-Canada; ASA24). Sub-sample contained those children whose parents indicated that their 24-hour recall represented typical intake.**

| **Beverage Group** | **Mean Beverage Volume NHQ in cups/day (SD)** | **Mean Beverage Volume ASA24 in cups/day (SD)** | **Mean Difference (NHQ – ASA24) cups/day (95% CI)** | **Wilcoxon Signed Rank Test of Difference** | **Spearman Correlation (95% CI)** |
| --- | --- | --- | --- | --- | --- |
| **Total SCBs** | 0.47 (0.75) | 0.37 (0.55) | 0.09 (-0.06, 0.24) | p=0.27 | 0.37 (0.19, 0.53) |
| **100% Juice** | 0.39 (0.68) | 0.14 (0.31) | 0.25 (0.13, 0.37) | p<0.0001 | 0.50 (0.34, 0.64) |
| **Sweetened Drinks + Soda or Pop** | 0.07 (0.29) | 0.23 (0.44) | -0.16 (-0.25, -0.06) | p=0.0004 | 0.19 (-0.003, 0.37) |

Beverage volumes are reported as mean (SD). Mean difference and Spearman correlation are reported with the 95% confidence interval (CI).
