# Supplementary material for: Validation of a parent proxy-reported beverage screener compared to a 24-hour dietary recall for the measurement of sugar-containing beverage intake among young children
Source: PLoS One. 2023 Jul 20;18(7):e0288768. doi: 10.1371/journal.pone.0288768 (PMC10358879; doi:10.1371/journal.pone.0288768)
Supplement: S3 Table — Sub-sample contained those children whose parents indicated that their 24-hour recall represented typical intake. Kappa statistic is reported with the 95% confidence interval (CI). (DOCX) [file pone.0288768.s004.docx]

**S3 Table: Kappa coefficient, sensitivity, and specificity for any daily SCB intake (>0 cups/day) compared to none (0 cups/day) for a sub-sample of N=101 children participating in the validation of a parent proxy-reported short beverage screener (Nutrition and Health Questionnaire; NHQ) against a 24-hour recall (Automated Self-Administered 24-h Dietary Assessment Tool-Canada; ASA24). Sub-sample contained those children whose parents indicated that their 24-hour recall represented typical intake.**

| **NHQ** | | **ASA24** | | **k (95% CI)** | **Sensitivity** | **Specificity** |
| --- | --- | --- | --- | --- | --- | --- |
|  |  | **Any** | **None** |  |  |  |
| **Total SCBs** | **Any** | 25 | 14 | 0.38 (0.20, 0.56) | 0.61 | 0.77 |
|  | **None** | 16 | 46 |  |  |  |
| **100% Juice** | **Any** | 17 | 19 | 0.45 (0.27, 0.63) | 0.81 | 0.76 |
|  | **None** | 4 | 61 |  |  |  |
| **Sweetened Drinks + Soda or Pop** | **Any** | 4 | 4 | 0.14 (-0.05, 0.33) | 0.16 | 0.95 |
|  | **None** | 21 | 72 |  |  |  |

Kappa statistic is reported with the 95% confidence interval (CI).
